# A rare case of pulmonary tuberculosis with simultaneous pulmonary and skin sarcoidosis: a case report

**DOI:** 10.1186/1757-1626-3-24

**Published:** 2010-01-13

**Authors:** Kornelija Mise, Ivana Goic-Barisic, Neira Puizina-Ivic, Igor Barisic, Marija Tonkic, Irena Peric

**Affiliations:** 1 Department of Pulmonary Diseases and School of Medicine University of Split, Split, Croatia; 2 Department of Clinical Microbiology and School of Medicine University of Split, Split, Croatia; 3 Department of Dermatology Diseases and School of Medicine University of Split, Split, Croatia; 4 Department of Diagnostic and Interventional Radiology and School of Medicine University of Split, Split University Hospital, 21000, Split, Croatia

## Abstract

**Background:**

Tuberculosis and sarcoidosis are chronic diseases that rarely occur concomitantly. Sarcoidosis is a multisystem granulomatous disorder characterized pathologically by the presence of non-caseating granulomas in involved tissues. Tuberculosis is infectious disease caused by *Mycobacterium tuberculosis *characterized by granulomas with caseous necrosis.

**Case presentation:**

We present a case of 43-year-old female refugee from Kosovo with microbiological confirmation of pulmonary tuberculosis and pulmonary and skin sarcoidosis at the same time. Three weeks after corticosteroid therapy for pulmonary sarcoidosis was introduced, positive finding of mycobacterium culture of bronchial aspirate was observed. Based on these results, corticosteroid therapy was excluded and antituberculous therapy was introduced for six months. In the meantime, new nodes on face and nose appeared and skin sarcoidosis was diagnosed. The patient was given corticosteroids and colchicine according to the skin and pulmonary sarcoidosis therapy recommendation.

**Conclusion:**

The authors of this study suggest that in cases when there is a dilemma in diagnosis between tuberculosis and sarcoidosis we should advance with corticosteroid therapy until we have microbiological confirmation of mycobacterium culture. This case is remarkable because this is a third described case of sarcoidosis and tuberculosis together (the first reported in Asia, the second in South Africa), and to authors knowledge, this is a first case report in Europe.

## Background

Tuberculosis and sarcoidosis are chronic granulomatous diseases. In many aspects, they are similar although different. Tuberculosis is infectious disease caused by *Mycobacterium tuberculosis *(MTB) characterized by granulomas with caseous necrosis, and the treatment is focused on elimination of microorganism. Sarcoidosis is systemic disease of unknown etiology, and is characterized by noncaseous granulomas, therefore it cannot be treated etiologically but immunosuppressive as a systemic immunological disease [1,2]. Overall mortality from sarcoidosis is 1-5% usually from respiratory, cardiac or central nervous system disease [3]. The histological similarity between sarcoidosis and tuberculosis (with epithelioid cell granulomas as the typical common finding) has stimulated the search for an association between mycobacterium and sarcoidosis, and it has been hypothesized that sarcoidosis could be a separate manifestation of infection with mycobacterium [2]. These diseases occur concomitantly very rarely [4,5]. Less commonly, tuberculosis develops as an opportunistic infection in patients following corticosteroid treatment for sarcoidosis.

## Case presentation

In this very rare case of 43 years-old female, refugee from Kosovo, was admitted as an outpatient to the Department of pulmonary diseases in 2003. because of erythema nodosum and dry cough. She was without fever during admission, smoked up to 30 cigarettes daily and was healthy most of her life. She suffered only of head injury and face trauma acquired during the war (during 1995.) by shelling. Three years before the outbreak of erythema nodosum, two small red subcutaneous tubercles were found on her forehead, in the left eyebrow area. The biopsy test showed chronic granulomatous infection that was described as a reaction to a foreign body.

**Figure 1:**
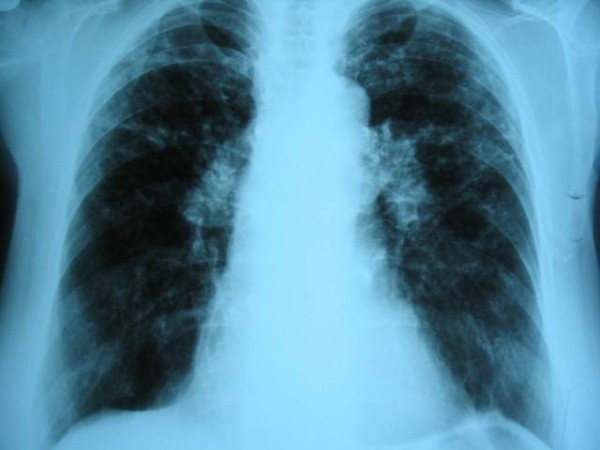
**Chest radiogram with fibroproductive changes in the area of both upper lobes and mycronodular interstitial infiltration bilateral**.

**Figure 2:**
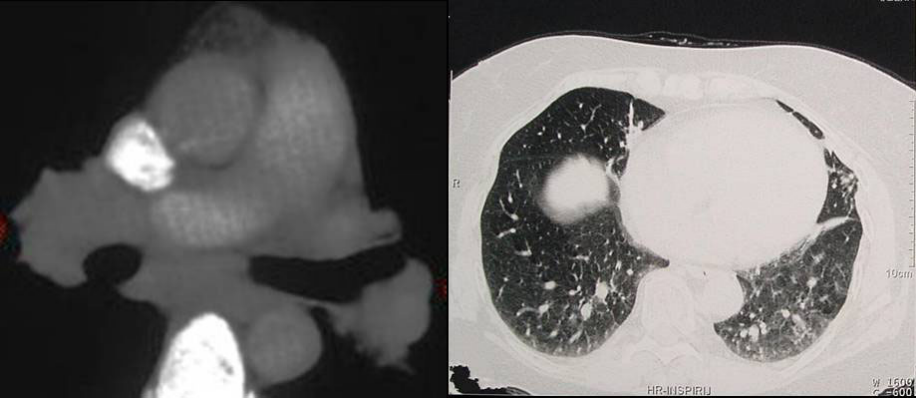
**Thorax CT with hilar lymph nodes enlargement (a) and reticular interstitial pattern with granulomas of both lower lobus (b)**.

**Figure 3:**
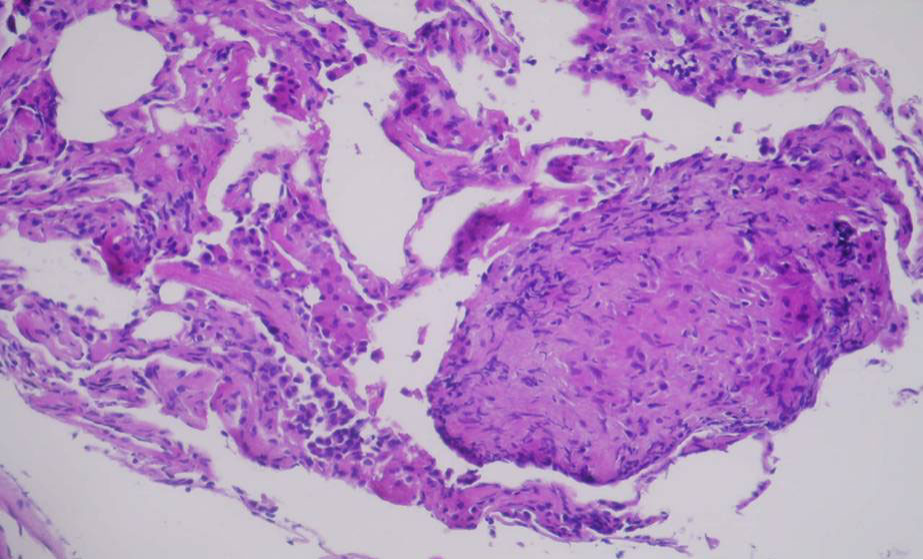
**Pathohistological findings of lung and bronchial walls biopsy showed chronic granuloma infection without caseotin necrosis, characteristic for sarcoidosis**.

**Figure 4:**
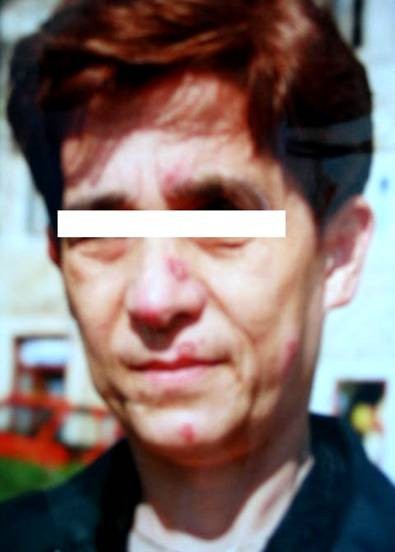
**Multiple new nodes on face during ATL therapy pathohistological verified as sarcoide granulomas**.

**Figure 5:**
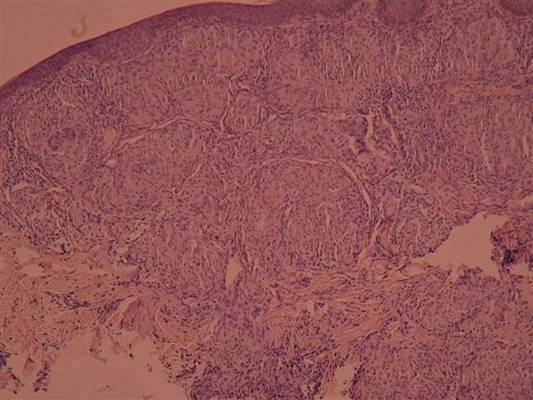
**Pathohistological evidence of noncaseating granulomas in skin biopsy, characteristic for sarcoidosis**.

Laboratory analysis revealed ESR 37 (normal range 0-15 mm/h), WBC 9.030/mm^3^, RBC 3,7/mm^3 ^and C-reactive protein 9.8 mg/L, AST 60 U/L, ALT 76 U/L, GGT 86 U/L, AP 79 U/L, while urea, creatinine, calcium in serum and 24 hours urine were within normal range. Angiotensin-converting enzyme (ACE) was elevated 108 U/l (normal 0-56). Tuberculosis test (2 IU) was 14 mm of induration in diameter. The result of spirometry measured 85% vital capacity (VC), 94,5% of forced vital capacity (FCV), 77% of forced expiratory volume (FEV),82,4% of forced expiratory volume in first second (FEV_1_) and FEV_1_/FCV 87,2% from referral values. Arterial blood gases were pO_2 _9,8 kPa, pH 7.45 and diffusing capacity of carbon monoxide (D_L_CO) 72%. Chest radiogram showed fibroindurative changes in the area of both upper lobes with lung traction. In upper lobes subpleurally and more to the left there was a focus with «round glass» pattern. Mycronodular interstitial infiltrations were found bilateral on chest radiogram with fibrous changes of hilus (Figure 1). High-resolution computed tomography (HRCT) of thorax showed interlobar and peribronchial thickness in upper lung lobes with subpleural fibroses and deformation of bronchi. There were minor areas of fresh infiltrations in apices on both sides, suggesting a new pathological process. There was a cavity 8 × 12 mm large in apical-posterior segment of the upper left lobe. Hilar lymph nodes were more then 2 cm in diameter, some of them had fibrous changes and some were calcificated. High resolution thorax CT with hilar lymph nodes enlargement and granulomas with reticular interstitial pattern in the lower respiratory part is present on the Figure 2(a, b). Fiberbronchoscopy revealed slightly edematous and hyperemic mucous membrane of bronchi on both sides, while purulent secrete was seen in bronchus left upper lobe and in lingul. The routine bacteriological culture of bronchial aspirate was sterile. Direct smear microscopy examination of the sample for the presence of acid-resistant bacilli by using Ziehl-Neelsen method was negative. Pathohistological findings of lung (Figure 3) and bronchial walls biopsy showed chronic granulomatous infection without caseous necroses. In bronchoalveolar lavage (BAL) CD4+ lymphocytes and alveolar macrophages were predominantly. The ratio of CD4+ and CD8+ lymphocytes was 5.5:1. All relative findings, including pathohistological examination indicate lung sarcoidosis. The patient was stared corticosteroid therapy (out of hospital-out-hospital patient) according to usual scheme for lung sarcoidosis, prednisolone 1 mg/kg body weight. Three weeks after introducing corticosteroid therapy, culturing the sample on Lowenstein-Jensen solid medium by using liquid culture method MGIT (Mycobacterium Growth Indicator Tube), *Mycobacterium tuberculosis *was isolated. The sensitivity test was performed by using the proportion method on solid Lowenstein-Jansen culture according to CLSI (Clinical and Laboratory Standards Institute) standards. All the four routine tested antituberculotics (isoniazid, rifampicin, ethambutol and streptomycin) responded well to sensitivity test and the susceptibility of pyrasinamide was confirmed in the referral national laboratory. A previous diagnosis was corrected to microbiological confirmed lung tuberculosis. Corticosteroid therapy was excluded after three weeks of treatment and antituberculotic therapy (ATL) was introduced. The patient was treated with rifampicin 600 mg, isoniazid 400 mg, etambuthol 1200 mg and pyrazinamide 1500 mg, on a daily basis during two months, after pyrazinamide was excluded and triple therapy was continued for one month. After three months from the beginning of ATL treatment, ethambuthol was excluded and rifampicin and isoniazid were continued for the next 3,5 months (entirely 6,5 months) with controlling regular hematological and biochemical analysis. The patient was in good clinical condition. However, ACE was continuously raised, 80-100 U/l. After four months of ATL therapy chest radiogram was made showing complete regression of tiny infiltrates in both upper lobes and small regression of nodular infiltrates which was described as unsatisfactory. During ATL therapy new nodes on face and nose, similar to those on the forehead appeared (Figure 4). The biopsy of skin changes was made and the pathohistological confirmation for sarcoidosis is evident on the Figure 5. Antituberculotic therapy was preceded for next two months until negative mycobacterium culture in new collected sputum was obtained. In course of the last month of TB therapy novel bigger, firm nodes appeared on ear lap. Pathohistological tests indicate sarcoidosis of the skin.

Microbiological examinations of the skin substrata in direct microscopy and polymerase chain reaction (PCR) with specific primer had not confirmed presence of *M. tuberculosis*. After 6 months of the beginning of ATL treatment, the patient received corticosteroid (prednisolone 40 mg daily) and colchicine therapy (200 mg daily) for lung and skin type of sarcoidosis. After four months of the corticosteroid therapy chest radiogram showed almost complete regression of nodular infiltrates in lung parenchyma. The skin changes were considerably smaller, dry on surface, and there were no new lesions on the skin while ACE was in normal rate (30 U/l).

## Discussion

A rare case of skin and lung sarcoidosis with positive mycobacterium culture obtained from bronchial aspirate has been presented. Sarcoidosis is a common multisystem granulomatous disease that frequently involves the lungs and can result in pulmonary fibrosis [2]. Based on positive finding of the bronchial aspirate, the diagnosis of lung TB was confirmed. According to authors' knowledge there has not been described a case with positive culture on MTB obtained from bronchial aspirate and with noncaseous granulomas in lung parenchyma, bronchial wall and skin. Pathohistological findings of lung and bronchial wall suggested on sarcoidosis, but confusing fact was positive tuberculosis skin test, although it can be positive in the cases of sarcoidosis. Recent innovative blood tests that measure the cell-mediated immune response of TB-infected individuals like quantiferon test are highly specific for detecting *M. tuberculosis *infection and may be helpful in diagnostic evaluations in conjuction with risk assessment and radiography [6]. At the time of patient admission, we did not use quantiferon-TB Gold test yet.

In the last 20 years, the research papers have recorded detection of mycobacterial DNA in some sarcoide lesions, especially in lymph nodes that indicates possible connection between these two diseases [7-11]. Silent asymptomatic stage I of sarcoidosis complicated with pulmonary tuberculosis has been recent published [12].

Sarcoidosis may presents with a wide range of symptoms. Noncaseating epitheloid cell granulomas characterizing sarcoidosis may affect most organs, including the skin. Skin lesions may be the only manifestations, or just one of several other organ involvements. Cutaneous lesions are present in ~25% of sarcoidosis patients [13]. The skin lesions could have specific aspects (papules, plaques, nodules, alopecia or purple scars) or can be more or less nonspecific (erythema nodosum) [14]. In the Netherlands epidemiological study sarcoidosis was mostly associated with rheumatoid arthritis [15].

The presented case has been of clinical interest because it happens very rarely that pulmonary tuberculosis and lung sarcoidosis coincide in the same patient with noncaseous lung granulomas. The dilemma was present only because Mantoux test (skin test) was positive. In the former states of Yugoslavia tuberculosis skin test was positive in more than 95% of population because bacillus *Calmette-Guerin *vaccination (BCG vaccine) was compulsory. It had to be carried out just after birth and then three times until a person was 18 years old. In spite of vaccination, inhabitants can come in touch with the infected persons, and it happened more often during the war in Croatia (1991.-1995.) and another parts of former Yugoslavia, because of the migration of the population from other parts of the Balkan (refugees, fugitives from Bosnia and Kosovo) where the incidence of lung tuberculosis was very high, comparing to Croatia (Kosovo 80/100000, Southern Croatia 18-20/100 000).

In the Sothern Croatia ratio of tuberculosis and sarcoidosis is 1:10 [16]. During ATL therapy complete regressions of new infiltrates in the upper lobe without mycronodular infiltrates has been achieved. Skin sarcoidosis was pathohistological confirmed, with negative direct smear microscopy examination in Ziehl-Neelsen stain and absence of mycobacterium DNA in skin granulomas.

Absence of mycronodular infiltrates (granulomas) in lung parenchyma and reduced changes of skin during 4 months of corticosteroid therapy have additionally proved the diagnosis of sarcoidosis with *M. tuberculosis *positive lung tuberculosis. Three years before sarciodosis and tuberculosis had been diagnosed, skin forehead granuloma was verified which was explained as granuloma of a foreign body due to previous injuries at shelling. However, according to this study, skin sarcoidosis cannot be excluded at that time. If the bronchial aspirate had not been sent for microbiological testing and if the therapy with corticosteroid had continued, a severe type of lung TB with cavities and probably galloping tuberculosis would have been developed. This particular represented case suggests us if suspicion of tuberculosis is high (for example when the patient had a tuberculin test of 14 mm, a cavity 8 × 12 mm in the apical-posterior segment of the upper left lobe and purulent secretion from the left upper lobe bronchus on bronchoscopy) the ATL treatment can start immediately. In the cases when patient's condition is rapidly deteriorating, corticosteroids and antituberculosis therapy could be started concurrently, until microbiological confirmation is established.

In cases when there is a dilemma in diagnosis between tuberculosis and sarcoidosis, we should advance with corticosteroid therapy until we have microbiological confirmation of mycobacterium culture [17]. In this particular case the doubt has been present from the very beginning, because of the positive skin test and data of previous living place where the incidence of tuberculosis is still high.

## Conclusion

We have presented the case of a patient with microbiological confirmation of pulmonary tuberculosis and pulmonary and skin sarcoidosis at the same time and have emphasized the diagnostic dilemmas that may occur when these conditions coexist. The beginning of steroid therapy in patients with underlying infection may accentuate life-threatening complications. The proper evaluation of all relevant findings and case-history data needs to be considered. The authors suggest that in cases when there is a dilemma in diagnosis between tuberculosis and sarcoidosis we should advance with corticosteroid therapy until we have microbiological confirmation of mycobacterium culture.

## Abbreviations

MTB: *Mycobacterium tuberculosis*; ESR: Erythrocyte sedimentation rate; WBC: white blood cell; RBC: red blood cell; AST: aspartate aminotransferase; ALT: alanine aminotransferase; GGT: gamma-glutamyl transferase; AP: alkaline phosphatase; ACE: Angiotensin-converting enzyme; VC: Vital capacity; FEV: Forced expiratory volume; FCV: forced vital capacity; FEV: forced expiratory volume; FEV_1_: forced expiratory volume in first second; D_L_CO: Diffusing capacity of carbon monoxide; HRCT: High-resolution computed tomography; BAL: Bronchoalveolar lavage; MGIT: Mycobacterium Growth Indicator Tube; ATL: Antituberculotic therapy; TB: Tuberculosis; PCR: Polymerase chain reaction.

## Consent

Written informed consent was obtained from the patient for publication of this case report and accompanying images. A copy of the written consent is available for review by editor-in-chief of this journal.

## Competing interests

The authors declare that they have no competing interests.

## Authors' contributions

KM conceived the study and participated in patient management, treated the patient and collected written informed consent. IGB participated in acquisition of data, interpretation of data and was major contributor in writing the manuscript. NPI and IB revised critically the manuscript adding substantial intellectual content. MT and IP provided data read and approved the manuscript. All aurhors read and approved the final manuscript.

## References

[B1] Hunningahkake GW Costabel U Ando M Baughman R Cordier JF du Bois R ATS/ERS/WASOG statement on sarcoidosis Sarcoidosis Vasc Diffuse Lung Dis 1999 16 149 173 10560120

[B2] Gal A Koss M The pathology of sarcoidosis Curr Opin Pulm Med 2002 8 445 451 10.1097/00063198-200209000-00018 12172451

[B3] Costabel U Hunninghake GW ATS/ERS/WASOG statement on Sarcoidosis Eur Respire J 1999 14 735 737 10.1034/j.1399-3003.1999.14d02.x 10573213

[B4] Wong CF Yew WW Wong PC Lee J A case of concomitant tuberculosis and sarcoidosis with mycobacterial DNA present in the sarcoid lesion Chest 1998 114 626 629 10.1378/chest.114.2.626 9726757

[B5] Oluboyo PO Awotedu AA Banach L Concomitant sarcoidosis in a patient with tuberculosis: first report of association in Africa Cent Afr J Med 2005 51 123 125 17447345

[B6] Mori T Sakatani M Yamagishi F Takashima T Kawabe Y Nagao K Shigeto E Harada N Mitarai S Okada M Suzuki K Inoue Y Tsuyuguchi K Sasaki Y Mazurek GH Tsuyuguchi I Specific detection of tuberculosis infection: an integron-gamma-based assay using new antigen Am J Respir Crit Care Med 2004 170 59 64 10.1164/rccm.200402-179OC 15059788

[B7] Giotaki HA Stefanou DG Biopsy documented tuberculous pleural effusion in a patient with biopsy-proven sarcoidosis Respiration 1988 54 193 196 10.1159/000195521 3247519

[B8] Milman N Lisby G Frus S Prolonged culture for mycobacteria in mediastinal lymph nodes from patients with pulmonary sarcoidosis Sarcoidosis Vas Diffuse Lung Dis 2004 21 25 28 15127971

[B9] Saboor SA Johnson NM Mc Fadden J Detection of mycobacterial DNA in sarcoidosis and tuberculosis with polymerase chain reaction Lancet 1992 339 1012 1015 10.1016/0140-6736(92)90535-B 1349051

[B10] Richter E Greinert U Kirsten D Rüsch-Gerdes S Schlüter C Duchrow M Galle J Magnussen H Schlaak M Flad HD Gerdes J Assessment of mycobacterial DNA in cells and tissue of mycobacterial and sarcoid lesions Am J Respir Crit Care Med 1996 153 375 380 8542146 10.1164/ajrccm.153.1.8542146

[B11] Fite E Fernandez-Figueras MT Prats R Vaguero M Morera J High prevalence of *Mycobacterium tuberculosis *DNA in biopsies from sarcoidosis patients from Catalonia, Spain Respiration 2006 73 20 26 10.1159/000087688 16113515

[B12] Papaetis GS Pefanis A Solomon S Tsangarakis I Orphanidou D Achimastos A Asymptomatic stage I sarcoidosis complicated by pulmonary tuberculosis: a case reports J Med Case Reports 2008 2 226 229 10.1186/1752-1947-2-226 18605996 PMC2474644

[B13] Judson MA Thompson BW Rabin DL Steimel J Knattereud GL Lackland DT Rose C Rand CS Baughman RP Teirstein AS The diagnostic pathway to sarcoidosis Chest 2003 123 406 412 10.1378/chest.123.2.406 12576358

[B14] Wilsher ML Seasonal clustering of sarcoidosis presenting with erythema nodosum Eur Respir J 1998 12 1197 1199 10.1183/09031936.98.12051197 9864021

[B15] Wirnsberger RM de Vries J Wouters EFM Drent M Clinical presentation of sarcoidosis in the Netherlands. An epidemiological study Neth J Med 1998 53 53 60 9766153 10.1016/s0300-2977(98)00058-8

[B16] Mise K Jankovic S Andjelinovic Š Ivancevic Z Forenpoher G Sarcoidosis in the area of south Croatia (Dalmatia-southest coast of Adriatic sea) Sarcoidosis Vasc Diffuse Dis 2001 18 60

[B17] Paramonthayan S Lasserson T Treatments for pulmonary sarcoidosis Respir Med 2008 102 1 9 10.1016/S0954-6111(08)70002-7 17954027

